# The interactions among factors associated with the risk of lung cancer among diabetes patients: a survival tree analysis

**DOI:** 10.1038/s41533-025-00417-x

**Published:** 2025-03-30

**Authors:** Sarah Tsz Yui Yau, Chi Tim Hung, Eman Yee Man Leung, Albert Lee, Eng Kiong Yeoh

**Affiliations:** JC School of Public Health and Primary Care, The Chinese University of Hong Kong, Hong Kong SAR, China

**Keywords:** Respiratory tract diseases, Epidemiology, Lifestyle modification, Preventive medicine

## Abstract

Past epidemiological studies demonstrated mixed findings on the association between diabetes and lung cancer. Given the possible links between diabetes, smoking, and respiratory diseases, this study aims to examine the interaction patterns among factors associated with the risk of lung cancer among diabetes patients. A territory-wide retrospective cohort study was performed using electronic health records of Hong Kong. Patients who received diabetes care in general outpatient clinics between 2010 and 2019 without cancer history were included and followed up until December 2019. Conditional inference survival tree was applied to examine the interaction patterns among factors associated with the risk of lung cancer. A total of 385,521 patients were included. During a median follow-up of 6.2 years, 3395 developed lung cancer. Age emerged as primary factor in differentiating the risk of lung cancer. Conditional on age ( ≤ 64 vs >64 years), smoking appeared as subsequent dominant risk factor within each subpopulation. Among old smokers aged >64 years characterized by long duration of diabetes (median: 6–8 years), chronic obstructive pulmonary disease (COPD) emerged as key risk factor. Six distinct subgroups of diabetes patients with different risk levels of lung cancer according to age, smoking, metformin use, and COPD status were identified. Findings of the study suggest the interaction patterns among age, smoking, and COPD on the risk of lung cancer among diabetes patients, providing targets for public health interventions.

## Introduction

Lung cancer is the most frequently diagnosed and deadliest cancer worldwide^1^. The major etiological factor is tobacco smoking. While diabetes is shown to be linked to several cancers^2,3^, its association with lung cancer remains inconclusive^2–6^.

Nevertheless, pathophysiological evidence suggests that under diabetes condition, the lung could be a target organ of microangiopathy, potentially increasing susceptibility to respiratory infections and pulmonary dysfunction^7^. Furthermore, in addition to diabetes, some factors associated with lung cancer such as smoking and chronic lung diseases^8^ may commonly contribute to intensified oxidative stress, chronic inflammation, and accelerated decline in pulmonary function beyond normal biological aging^9–11^, potentially elevating the risk of lung cancer.

While biological evidence suggests the possible intricate links among factors potentially associated with the risk of lung cancer, there is a lack of epidemiological studies examining how different factors interact to influence the risk of lung cancer among diabetes patients. The conflicting overall associations between diabetes and lung cancer found in previous studies^2–6^ could be potentially due to differential links by smoking^12^ or lung disease status^13^. Moreover, while biological aging is a risk factor for many cancers, it remains less clear how the concurrent presence of diabetes and possible weakened pulmonary function in diabetes^7^ may influence the risk of lung cancer among diabetes patients across different age groups by smoking status. Furthermore, it remains uncertain whether the presence of pulmonary diseases may individually^14^ or collectively^7,13^ influence the risk of lung cancer in the presence of other conditions.

Conditional inference survival tree^15^ is a tree-structured (or recursive partitioning) algorithm embedded with statistical theory in its partitioning procedures. Tree-structured algorithms are able to (i) capture interaction patterns among covariates^15,16^; and (ii) provide an intuitive approach for interpretation. Compared to other tree-structured algorithms, conditional inference survival tree has the advantages of (i) incorporating a theoretical framework; (ii) preventing overfitting; (iii) minimizing selection bias towards covariates with many possible values; and (iv) not requiring explicit pruning.

To fill the gap in the literature on the lack of epidemiological studies on how different combinations of factors are associated with the risk of lung cancer among diabetes patients, this study seeks to examine the interaction patterns among factors potentially associated with the risk of lung cancer among diabetes patients using a survival tree analysis approach.

## Methods

### Study design and study population

This is a territory-wide retrospective cohort study based on electronic health records of Hong Kong’s public healthcare system. The Hospital Authority (HA) is a statutory body responsible for managing 43 hospitals, 49 specialist outpatient clinics and 74 general outpatient clinics. The HA systematically stores records on patients’ demographics, disease diagnoses, prescription records, laboratory results, inpatient admission and outpatient attendance in a centralized data repository. Disease diagnoses were coded with the International Classification of Diseases, 9th or 10th revision (ICD-9 or ICD-10) or the International Classification of Primary Care 2nd edition (ICPC-2). Data were accessed via HA Data Collaboration Lab. Ethics approval for secondary data analysis was provided by the Chinese University of Hong Kong – Survey and Behavioural Research Ethics Committee (reference number: SBRE-22-0386).

### Patients

Patients who were diagnosed with diabetes and received a first diabetes complication screening assessment at any of the general outpatient clinics between 2010 and 2019 were initially included. Index date was defined as date of the first assessment. Those who (i) were diagnosed with non-type 2 diabetes; (ii) had missing information on time of diabetes diagnosis; (iii) received a diabetes diagnosis below the age of 18 years; (iv) had a history of malignancy; or (v) had a follow-up period of less than six months were excluded. Patients were followed up until a lung cancer diagnosis, death, or December 2019, whichever occurred earlier.

### Outcome

The outcome of interest was diagnosis of lung cancer (ICD-9: 162; ICD-10: C33-34) during follow-up.

### Covariates

Information on input variables was ascertained during the first assessment. Candidate split covariates included demographics (age and sex), disease history, medication use, behavioral factors, anthropometric and laboratory measurements. Disease history included duration of diabetes, family history of diabetes, lung diseases (chronic obstructive pulmonary disease [COPD]^8^ and pneumonia)^17^, and common comorbidities (ischemic heart disease, cerebrovascular disease, heart failure, hypertension, chronic kidney disease, and liver cirrhosis). Medication use included commonly used anti-diabetic drugs (metformin, sulfonylurea, insulin, and dipeptidyl peptidase-4 inhibitors), aspirin, non-steroidal anti-inflammatory drugs, anti-coagulants, anti-platelets, anti-hypertensive drugs, and statins. Medication use was defined as whether a patient had been prescribed a drug at the time of assessment. Behavioral factors included smoking and alcohol use. Anthropometric measurements included body mass index and waist-to-hip ratio. Laboratory measurements included HbA_1c_, fasting glucose, low-density lipoprotein cholesterol, high-density lipoprotein cholesterol, triglycerides, and serum creatinine. Laboratory measurements were taken from results closest to the time of the assessment within one year.

### Data analysis

Conditional inference survival tree^15^ was applied to examine the interaction patterns among factors associated with the risk of lung cancer. At each split, a global null hypothesis of independence between a set of covariates and the outcome was tested at a pre-determined α level. If rejected, a set of partial null hypotheses of independence between each covariate and the outcome were tested at the same α level. The covariate with the strongest association or smallest Bonferroni-corrected p-value was then chosen as split variable. The algorithm recursively conducted partitioning until the global null hypothesis cannot be rejected. The α level and maximum depth of the tree model were set at 0.01 and 4 respectively. For continuous split variables, the cutoff value was selected to optimize (maximize) differences in between-group survival outcomes. Each path from the root node to a terminal node represented an interaction pattern^16^, where the effects of a split variable were conditional on split variables selected at its ancestor nodes. Patients were separated into mutually exclusive subgroups of most homogenous within-group survival outcomes at terminal nodes. The cumulative lung cancer incidence during follow-up across identified distinct subgroups was graphically examined. Model performance was assessed using area under the curve (AUC) as metric. In post-hoc analyses, Cox proportional hazards regression was applied to examine the association between each identified important factor and the risk of lung cancer.

## Results

**Fig. 1:**
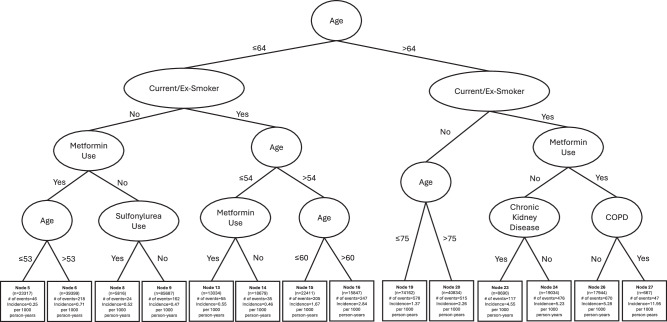
Survival tree diagram for lung cancer incidence among diabetes patients. COPD chronic obstructive pulmonary disease.

Of the 385,521 patients included, 3395 patients developed lung cancer during a median follow-up of 6.2 years. The incidence rates among smokers and non-smokers were 2.93 and 1.00 per 1000 person-years respectively. In the tree model, age emerged as primary factor in differentiating the risk of lung cancer. Conditional on age (≤64 vs >64 years), smoking appeared as subsequent predominant risk factor within each age-specific subpopulation. Among old smokers aged >64 years who took metformin with longer duration of diabetes (median: 6–8 years), COPD emerged as important risk factor. Six distinct subgroups of patients were identified, namely young never smoker, young ever smoker, old never smoker, old ever smoker without metformin use, old ever smoker in the absence of COPD with metformin use, and old ever smoker in the presence of COPD and metformin use (Fig. 1; Table 1).

**Table 1:** Characteristics of distinct subgroups of diabetes patients.

	Young Never Smoker	Young Ever Smoker	Old Never Smoker	Old Ever Smoker short diabetes history	Old Ever Smoker long diabetes history absence of COPD	Old Ever Smoker long diabetes history presence of COPD
	Node {5,6,8,9}	Node {13,14,15,16}	Node {19,20}	Node {23,24}	Node 26	Node 27
Characteristics	(n = 154,219)	(n = 69,971)	(n = 114,996)	(n = 27,724)	(n = 17,944)	(n = 667)
Number of lung cancer cases during follow-up, n (%)	450 (0.29%)	542 (0.77%)	1093 (0.95%)	593 (2.14%)	670 (3.73%)	47 (7.05%)
Demographics						
Male, n (%)	58,786 (38.12%)	62,827 (89.79%)	33,319 (28.97%)	25,231 (91.01%)	15,903 (88.63%)	586 (87.86%)
Age at assessment in year, mean ± SD	54.79 ± 7.33	54.07 ± 7.56	73.48 ± 6.48	72.98 ± 6.38	72.88 ± 6.07	75.66 ± 6.48
Duration of diabetes in year, median (IQR)	2 (0–7)	2 (0–7)	5 (1–12)	3 (1–9)	8 (3–13)	6 (2–11)
Disease history						
COPD, n (%)	75 (0.05%)	419 (0.60%)	484 (0.42%)	1224 (4.41%)	0 (0%)	667 (100%)
Pneumonia, n (%)	2471 (1.60%)	1893 (2.71%)	4583 (3.99%)	2114 (7.63%)	845 (4.71%)	220 (32.98%)
Ischemic heart disease, n (%)	5228 (3.39%)	5773 (8.25%)	10,442 (9.08%)	4414 (15.92%)	2163 (12.05%)	111 (16.64%)
Cerebrovascular disease, n (%)	4833 (3.13%)	3650 (5.22%)	9939 (8.64%)	3558 (12.83%)	1869 (10.42%)	86 (12.89%)
Heart failure, n (%)	1275 (0.83%)	1191 (1.70%)	3432 (2.98%)	1370 (4.94%)	487 (2.71%)	97 (14.54%)
Hypertension, n (%)	123,163 (79.86%)	54,876 (78.43%)	107,245 (93.26%)	25,338 (91.39%)	16,684 (92.98%)	612 (91.75%)
Chronic kidney disease, n (%)	19,777 (12.82%)	11,907 (17.02%)	18,257 (15.88%)	8690 (31.34%)	0 (0%)	0 (0%)
Liver cirrhosis, n (%)	3294 (2.14%)	1749 (2.50%)	1758 (1.53%)	566 (2.04%)	259 (1.44%)	18 (2.70%)
Family history of diabetes, n (%)	88,703 (57.52%)	38,700 (55.31%)	40,099 (34.87%)	8203 (29.59%)	5948 (33.15%)	159 (23.84%)
Medication use						
Anti-diabetic drugs						
Metformin, n (%)	62,716 (40.67%)	28,274 (40.41%)	47,103 (40.96%)	0 (0%)	17,944 (100%)	667 (100%)
Sulfonylurea, n (%)	37,989 (24.63%)	18,384 (26.27%)	34,165 (29.71%)	3368 (12.15%)	10,395 (57.93%)	347 (52.02%)
Insulin, n (%)	8804 (5.71%)	5257 (7.51%)	6518 (5.67%)	2269 (8.18%)	1149 (6.40%)	38 (5.70%)
Dipeptidyl peptidase-4 inhibitors, n (%)	5401 (3.50%)	2851 (4.07%)	4424 (3.85%)	1510 (5.45%)	604 (3.37%)	11 (1.65%)
Sodium-glucose cotransporter-2 inhibitors, n (%)	491 (0.32%)	273 (0.39%)	184 (0.16%)	95 (0.34%)	0 (0%)	0 (0%)
Glucagon-like peptide-1 receptor agonists, n (%)	119 (0.08%)	59 (0.08%)	16 (0.01%)	2 (0.01%)	2 (0.01%)	0 (0%)
Glucosidase inhibitors, n (%)	571 (0.37%)	251 (0.36%)	531 (0.46%)	50 (0.18%)	164 (0.91%)	2 (0.30%)
Meglitinide, n (%)	64 (0.04%)	20 (0.03%)	44 (0.04%)	9 (0.03%)	9 (0.05%)	0 (0%)
Glitazone, n (%)	677 (0.44%)	234 (0.33%)	356 (0.31%)	46 (0.17%)	100 (0.56%)	2 (0.30%)
Any of the above, n (%)	79,138 (51.32%)	37,630 (53.78%)	61,436 (53.42%)	6797 (24.52%)	17,944 (100%)	667 (100%)
Aspirin, n (%)	18,322 (11.88%)	13,894 (19.86%)	31,721 (27.58%)	10,579 (38.16%)	5748 (32.03%)	261 (39.13%)
Non-steroidal anti-inflammatory drugs, n (%)	85,813 (55.64%)	36,126 (51.63%)	63,188 (54.95%)	14,120 (50.93%)	8147 (45.40%)	323 (48.43%)
Anti-coagulants, n (%)	4096 (2.66%)	4129 (5.90%)	6733 (5.85%)	2927 (10.56%)	1067 (5.95%)	52 (7.80%)
Anti-platelets, n (%)	6250 (4.05%)	5646 (8.07%)	9926 (8.63%)	5882 (21.22%)	0 (0%)	0 (0%)
Anti-hypertensive drugs, n (%)	93,567 (60.67%)	41,278 (58.99%)	93,163 (81.01%)	21,248 (76.64%)	15,009 (83.64%)	526 (78.86%)
Statins, n (%)	69,586 (45.12%)	33,855 (48.38%)	62,660 (54.49%)	17,017 (61.38%)	8322 (46.38%)	262 (39.28%)
Behaviors						
Current smoker/ ex-smoker, n (%)	0 (0%)	69,971 (100%)	0 (0%)	27,724 (100%)	17,944 (100%)	667 (100%)
Current drinker/ ex-drinker, n (%)	31,639 (20.52%)	41,218 (58.91%)	14,687 (12.77%)	14,557 (52.51%)	9420 (52.50%)	321 (48.13%)
Anthropometric measurements						
Body mass index in kg/m^2^, mean ± SD	26.58 ± 4.48	26.57 ± 4.30	25.43 ± 3.79	25.37 ± 3.51	25.02 ± 3.43	24.40 ± 3.76
Waist-to-hip ratio, mean ± SD	0.93 ± 0.06	0.95 ± 0.06	0.94 ± 0.07	0.96 ± 0.06	0.96 ± 0.06	0.97 ± 0.07
Laboratory measurements						
HbA_1c_ in %, mean ± SD	7.43 ± 1.50	7.63 ± 1.72	7.15 ± 1.20	7.21 ± 1.37	7.34 ± 1.30	7.20 ± 1.19
Fasting glucose in mmol/L, mean ± SD	7.77 ± 2.34	7.92 ± 2.58	7.34 ± 1.95	7.38 ± 2.08	7.39 ± 2.02	6.95 ± 1.94
Low-density lipoprotein cholesterol in mmol/L, mean ± SD	2.77 ± 0.83	2.69 ± 0.83	2.61 ± 0.82	2.48 ± 0.79	2.57 ± 0.77	2.65 ± 0.80
High-density lipoprotein cholesterol in mmol/L, mean ± SD	1.29 ± 0.33	1.16 ± 0.30	1.33 ± 0.35	1.22 ± 0.33	1.21 ± 0.32	1.32 ± 0.38
Triglycerides in mmol/L, mean ± SD	1.64 ± 1.19	1.84 ± 1.60	1.50 ± 0.89	1.50 ± 0.96	1.43 ± 0.90	1.34 ± 0.92
Serum creatinine in µmol/L, mean ± SD	73.70 ± 37.44	84.70 ± 46.42	84.02 ± 39.01	101.78 ± 55.22	93.56 ± 23.46	93.81 ± 28.52

*COPD* chronic obstructive pulmonary disease.

### Age and smoking

Age at 64 years was identified as primary factor in differentiating the risk of lung cancer among the overall diabetes population. Across the old (>64 years) and young (≤64 years) subpopulations, smoking symmetrically emerged as most dominant risk factor for lung cancer. Among old and young patients, ever smokers were 3.12 and 3.24 times likely to develop lung cancer respectively, when compared to never smokers, controlling for age, sex, and duration of diabetes (Table 2).

**Table 2:** Significant differences in adjusted hazard ratios of selected split variables between sibling or comparison nodes.

Node	Characteristics	Comparison i, COPD in old smoker with metformin use	Comparison ii, Smoking in young patients	Comparison iii, Smoking in old patients	Comparison iv, Distinct subgroups	Comparison v, Distinct subgroups
Node 26	Ever smoker aged >64 years in the absence of COPD with metformin use	1				
Node 27	Ever smoker aged >64 years in the presence of COPD with metformin use	2.09 (1.55–2.82)				
Node {5,6,8,9}	Never smoker aged ≤64 years		1			
Node {13,14,15,16}	Ever smoker aged ≤64 years		3.24 (2.77–3.80)			
Node {19,20}	Never smoker aged >64 years			1		
Node {23,24,26,27}	Ever smoker aged >64 years			3.12 (2.81–3.46)		
Node {5,6,8,9}	Young Never Smoker				1	1
Node {13,14,15,16}	Young Ever Smoker				2.83 (2.48–3.24)	2.83 (2.48–3.24)
Node {19,20}	Old Never Smoker				1.30 (1.12–1.50)	1.30 (1.12–1.50)
Node {23,24}	Old Ever Smoker with short diabetes history				4.10 (3.48–4.83)	4.10 (3.48–4.83)
Node 26	Old Ever Smoker with long diabetes history and absence of COPD				4.10 (3.50–4.81)	4.11 (3.50–4.81)
Node 27	Old Ever Smoker with long diabetes history and presence of COPD				8.33 (6.04–11.50)	8.38 (6.07–11.57)

Comparisons i to iv were made with adjustment of age, sex, and duration of diabetes. Comparison v was made with additional adjustment of HbA_1c_ and fasting glucose.

Adjusted hazard ratios are presented with 95% confidence interval.

*COPD* chronic obstructive pulmonary disease.

### Age, smoking, metformin use, and COPD

**Fig. 2:**
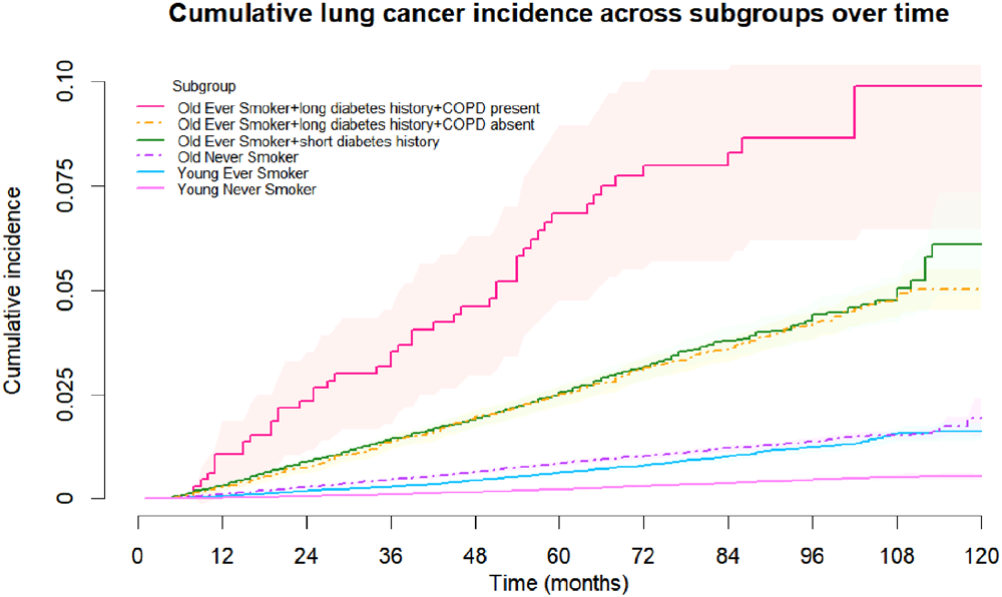
Cumulative lung cancer incidence across distinct subgroups of diabetes patients. COPD chronic obstructive pulmonary disease.

Among old ever smokers, the presence of COPD emerged as key factor in differentiating the risk of lung cancer in metformin users (Fig. 1). Those with COPD were 2.09 times likely to develop lung cancer when compared to those without COPD, adjusting for age, sex, and duration of diabetes (Table 2). Results remained similar when HbA_1c_ and fasting glucose were additionally controlled. Old ever smokers who took metformin and suffered from COPD had the highest risk of lung cancer development (Table 2; Fig. 2). They were also more likely to have a history of pneumonia when compared to other old ever smokers (33 vs 5–8%) (Table 1).

Among old ever smokers, metformin users tended to have a longer duration of diabetes (median: 6–8 vs 3 years) and were also more likely to be prescribed with sulfonylurea (52–58 vs 12%) when compared to metformin non-users (Table 1). Nevertheless, metformin use did not appear to be individually associated with the risk of lung cancer (Supplementary Table S1).

Overall, when compared to young never smokers, old never smokers, young ever smokers, old ever smokers without metformin use/COPD, and old ever smokers with metformin use and COPD were 1.30, 2.83, 4.10–4.11, and 8.38 times likely to develop lung cancer, when controlling for age, sex, duration of diabetes, HbA_1c_, and fasting glucose (Table 2).

### Model performance

The AUCs of the tree model at 2, 5, and 7 years were 0.764 (95%CI: 0.763–0.765), 0.743 (95%CI: 0.742–0.744), and 0.735 (95%CI: 0.734–0.736) respectively.

## Discussion

The present study revealed that despite tobacco smoking being a well-established risk factor for lung cancer, age, smoking, and COPD may interact to differentiate the risk of lung cancer in diabetes. The tree model identified age at 64 years as optimal age cutoff to primarily differentiate the risk of lung cancer among the study diabetes population. Among old ever smokers characterized by a longer history of diabetes (median: 6–8 years), the presence of COPD emerged as key risk factor for lung cancer. Young never smokers, old never smokers, young ever smokers, old ever smokers without metformin use/COPD, and old ever smokers with metformin use and COPD demonstrated a gradient of increasing lung cancer risk.

In the current study, age, smoking, and COPD exhibited an interaction pattern on the risk of lung cancer under diabetes condition. Aging, smoking, diabetes, and COPD are intricately linked and may collectively influence the risk of lung cancer among diabetes patients. Diabetes^18^ and COPD^19,20^ are both age-related diseases^18–20^, and characterized by chronic low-grade inflammation^18,20^ and cellular senescence^18,19,21^. Prior research has shown that COPD, but not asthma, is associated with an elevated risk of diabetes, potentially due to the shared oxidative stress, systematic inflammation mechanism, and cytokine profile between two diseases^22^. Elevated levels of proinflammatory factors in COPD may promote insulin resistance over time^22,23^. On the other hand, while smoking exposure is a major risk factor for COPD, it is estimated that one-third of patients with COPD are never smokers^24^. Previous research has demonstrated that COPD is a risk factor for lung cancer regardless of smoking status^25^. Nevertheless, smoking exposure may induce additional damage to the lungs by intensifying oxidative stress and triggering systematic inflammation^10^. Moreover, the repairing process of injured lungs may cause scar formation^10^. Smoking and COPD may both accelerate lung functioning decline faster than the normal physiological aging process^11^. In addition, cumulative exposure to carcinogens in tobacco smoke may increase with age. Furthermore, under chronic hyperglcemia, the lungs may suffer from further injury due to microangiopathy in diabetes^7^. As a result, in addition to direct exposure to carcinogens in tobacco smoke, the co-existence of accelerated decline in lung functioning and systematic inflammation under smoking exposure, chronic lung disease, and diabetes^10,22^, may collectively accelerate carcinogenesis of the lungs.

There are some potential public health implications of the present study. While the overall association between diabetes and lung cancer remains controversial in the literature^2–6^, biological aging, smoking, and COPD may collectively promote chronic inflammation and accelerate decline in lung functioning faster than normal aging in diabetes^11^. Prior research suggests that improved metabolic health may potentially delay progression of chronic lung diseases such as COPD^23^. In addition to preventing tobacco use, improved metabolic health may slow down deterioration of lung functioning in the presence of chronic lung diseases^23^, potentially lowering the risk of developing lung cancer under diabetes condition.

Some limitations are potentially present in the current study. First, information on cumulative exposure to active smoking was not available in this study. Dose-response effects of smoking were not evaluated. Second, smoking information was self-reported and prone to social desirability bias. Third, information on COPD severity was not available in this study. Fourth, glycemic levels were measured at baseline in this study. The subsequent change in glycemic levels was not captured. Fifth, dosage and duration of medication use was not evaluated in the present study. Sixth, information on occupational and environmental exposures to potential carcinogenic agents was not available in this study. Seventh, histological subtypes of lung cancer were not differentiated in the study. Lastly, the dominant factors and optimal cutoff for age may vary across different populations. Further studies are warranted to verify generalizability of the findings in other populations.

## Conclusions

This study suggests the interaction patterns among age, smoking, and COPD on the risk of lung cancer among diabetes patients. While tobacco smoking is a known risk factor for lung cancer, the concurrent presence of smoking, COPD, and diabetes on the background of biological aging may exhibit an interaction pattern on the risk of lung cancer. Findings of the study may help identify target groups for public health prevention strategies, and provide evidence for the importance of tobacco prevention and chronic lung disease management in attenuating the risk of lung cancer under age-associated conditions.

## Supplementary information

Supplementary Tables

## Data Availability

Data is not available for sharing due to access restriction.

## Code availability

The underlying code for this study is not publicly available but may be made available to qualified researchers on reasonable request from the corresponding author.
