# Supplementary material for: The interactions among factors associated with the risk of lung cancer among diabetes patients: a survival tree analysis
Source: NPJ Prim Care Respir Med. 2025 Mar 30;35:20. doi: 10.1038/s41533-025-00417-x (PMC11955524; doi:10.1038/s41533-025-00417-x)
Supplement: Supplementary file 1 — Supplementary Tables [file 41533_2025_417_MOESM1_ESM.docx]

Table S1. Non-significant differences in adjusted hazard ratios of selected split variables between sibling nodes.

| **Node** | **Characteristics** | **Comparison i,**  **Age in**  **young non-smoker**  **with metformin use** | **Comparison ii,**  **Sulfonylurea use in**  **young non-smoker**  **without metformin use** | **Comparison iii,**  **Metformin use in**  **youngest smoker** | **Comparison iv,**  **Age in**  **middle-old smoker** | **Comparison v,**  **Age in**  **old non-smoker** | **Comparison vi,**  **CKD in**  **old smoker**  **without metformin use** |
| --- | --- | --- | --- | --- | --- | --- | --- |
| Node 5 | Never smoker aged ≤53 years with metformin use | 1 |  |  |  |  |  |
| Node 6 | Never smoker aged between 54 to 64 years with metformin use | 1.42 (0.86-2.37) |  |  |  |  |  |
| Node 8 | Never smoker aged ≤64 years who took sulfonylurea but not metformin |  | 1 |  |  |  |  |
| Node 9 | Never smoker aged ≤64 years without metformin/sulfonylurea use |  | 1.23 (0.79-1.92) |  |  |  |  |
| Node 13 | Ever smoker aged ≤54 years with metformin use |  |  | 1 |  |  |  |
| Node 14 | Ever smoker aged ≤54 years without metformin use |  |  | 1.11 (0.71-1.73) |  |  |  |
| Node 15 | Ever smoker aged between 55 to 60 years |  |  |  | 1 |  |  |
| Node 16 | Ever smoker aged between 61 to 64 years |  |  |  | 1.38 (0.95-1.99) |  |  |
| Node 19 | Never smoker aged between 65 to 75 years |  |  |  |  | 1 |  |
| Node 20 | Never smoker aged >75 years |  |  |  |  | 1.12 (0.90-1.39) |  |
| Node 23 | Ever smoker aged >64 years in the presence of CKD without metformin use |  |  |  |  |  | 1 |
| Node 24 | Ever smoker aged >64 years in the absence of CKD without metformin use |  |  |  |  |  | 1.03 (0.84-1.28) |

CKD, chronic kidney disease.

Note 1: Comparisons i to ix were made with adjustment of age, sex, and duration of diabetes.

Note 2: Adjusted hazard ratios are presented with 95% confidence interval.

Table S1. Non-significant differences in adjusted hazard ratios of selected split variables between sibling nodes (continued).

| **Node** | **Characteristics** | **Comparison vii,**  **Metformin use**  **in young non-smoker** | **Comparison viii,**  **Age**  **in young smoker** | **Comparison ix,**  **Metformin use**  **in old smoker** |
| --- | --- | --- | --- | --- |
| Node {5,6} | Never smoker aged ≤64 years with metformin use | 1 |  |  |
| Node {8,9} | Never smoker aged ≤64 years without metformin use | 1.06 (0.87-1.30) |  |  |
| Node {13,14} | Ever smoker aged ≤54 years |  | 1 |  |
| Node {15,16} | Ever smoker aged between 55 to 64 years |  | 1.20 (0.83-1.73) |  |
| Node {23,24} | Ever smoker aged >64 years without metformin use |  |  | 1 |
| Node {26,27} | Ever smoker aged >64 years with metformin use |  |  | 1.06 (0.94-1.18) |

Note 1: Comparisons i to ix were made with adjustment of age, sex, and duration of diabetes.

Note 2: Adjusted hazard ratios are presented with 95% confidence interval.
